# Structural and functional characterization of a novel scFv anti-HSP60 of *Strongyloides* sp.

**DOI:** 10.1038/srep10447

**Published:** 2015-05-21

**Authors:** Marcelo Arantes Levenhagen, Fabiana de Almeida Araújo Santos, Patrícia Tiemi Fujimura, Ana Paula Caneiro, Julia Maria Costa-Cruz, Luiz Ricardo Goulart

**Affiliations:** 1 Laboratório de Diagnóstico de Parasitoses, Instituto de Ciências Biomédicas, Universidade Federal de Uberlândia, Uberlândia, Minas Gerais, Brazil; 2 Laboratório de Nanobiotecnologia, Instituto de Genética e Bioquímica, Universidade Federal de Uberlândia, Uberlândia, Minas Gerais, Brazil; 3 Department of Medical Microbiology and Immunology, University of California, Davis, California, United States of America

## Abstract

Phage display is a powerful technology that selects specific proteins or peptides to a target. We have used Phage Display to select scFv (single-chain variable fragment) clones from a combinatorial library against total proteins of *Strongyloides venezuelensis*. After scFv characterization, further analysis demonstrated that this recombinant fragment of antibody was able to bind to an *S. venezuelensis* antigenic fraction of ~65 kDa, present in the body periphery and digestive system of infective larvae (L3), as demonstrated by immunofluorescence. Mass spectrometry results followed by bioinformatics analysis showed that this antigenic fraction was a heat shock protein 60 (HSP60) of *Strongyloides* sp. The selected scFv was applied in serodiagnosis by immune complexes detection in serum samples from individuals with strongyloidiasis using a sandwich enzyme-linked immunosorbent assay (ELISA), showing sensitivity of 97.5% (86.84–99.94), specificity of 98.81 (93.54–99.97), positive likelihood ratio of 81.60 and an area under the curve of 0.9993 (0.9973–1.000). Our study provided a novel monoclonal scFv antibody fragment which specifically bound to HSP60 of *Strongyloides* sp. and was applied in the development of an innovative serodiagnosis method for the human strongyloidiasis.

Human strongyloidiasis is a neglected condition with worldwide distribution^1^,^2^. Immunocompetent individuals usually have a self-limited infection, but the parasite may remain in the body of the individual for up to 10 years without causing infection or being diagnosed. In individuals with immunosuppression, the infection may become life threatening due to hyperinfection and parasite spread to other parts of the body^3^,^4^.

The major problem for the serodiagnosis of human strongyloidiasis is caused the difficulty in obtaining larvae of *Strongyloides stercoralis* for antigen preparation. Because of this difficulty, heterologous antigens from *Strongyloides venezuelensis* have been used for convenience, due to similarities in transcripts that have key roles in the host-parasite interaction, as well as important molecules for diagnosis such as excretory/secretory proteins. Furthermore, a crude extract is routinely used, which results in cross-reactions with other parasitic infections^5^.

Phage display is a method which provides the selection of peptides, antibodies or single-chain variable fragment (scFv), expressed on bacteriophages, by standard methods with shorter time of production and important applicability in diagnosis due high specificity of the selected molecule to the target^6^,^7^,^8^,^9^. An scFv or specific-target antibody fragments represents the smallest functional heavy (VH) and light (VL) chains domain of an antibody^10^,^11^.

Here we report a strategy to select scFv clones from a combinatorial phage library against *Strongyloides venezuelesis* total proteins. The structure of the selected scFv and its binding was characterized by bioinformatics tools and the functionality of this novel scFv was characterized by development of a new serodiagnosis method.

## Results

### Selection, DNA sequencing, bioinformatic in silico analysis and purification of scFv

**Figure 1:**
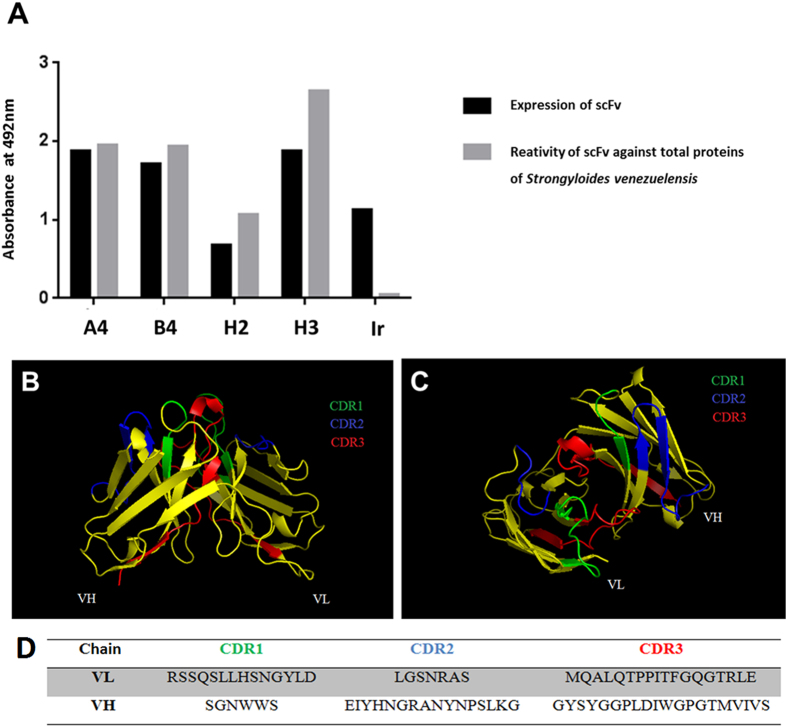
Expression and reactivity of scFv clones by ELISA at 492 nm and 3D structure of scFv. **** (**A**) Expression of scFv clones induced with isopropyl β-D-thiogalactopiranoside (IPTG) after *Escherichia coli* TOP10 transformation and reactivity of scFv clones against *Strongyloides venezuelensis* total proteins. (**B**) The 3D structure of the scFv molecule and (**C**) the predicted antigen-binding site, both analyzed by the PyMOL online tool. (**D**) Deduced amino acid sequence from CDR regions. Ir: Irrelevant scFv, CDR: complementarity determining region; VH and VL: heavy and light chains, respectively.

After two cycles of selection against *S. venezuelensis* total proteins, the selection efficiency was determined. Four out of 96 scFv clones that were expressed have bound to total proteins of *S. venezuelensis* as demonstrated by ELISA values, and clones were named A4, B4, H2 and H3 (Fig. 1A). The nucleotide sequences obtained were subjected to the IgBLAST program to obtain their amino acid sequences and characterize the scFv light and heavy chains, considering both the conserved framework regions (FR1, FR2 and FR3) and the variable complementarity determining regions (CDR1, CDR2 and CDR3). The four clones presented the same amino acid sequence, which was submitted to the Raptor x and PyMOL programs, which are in silico prediction tools, to obtain its 3D structure and identify the CDR regions (Fig. 1B,C). After medium-scale production of the selected clone, the scFv molecules were purified by HPLC over a His-Trap column. Two milliliters of scFv (750 μg/mL) were obtained. The dot blot assay confirmed the expression and efficiency of scFv purification.

### Pull-down assay, immunofluorescence antibody test (IFAT) and mass spectrometry

**Figure 2:**
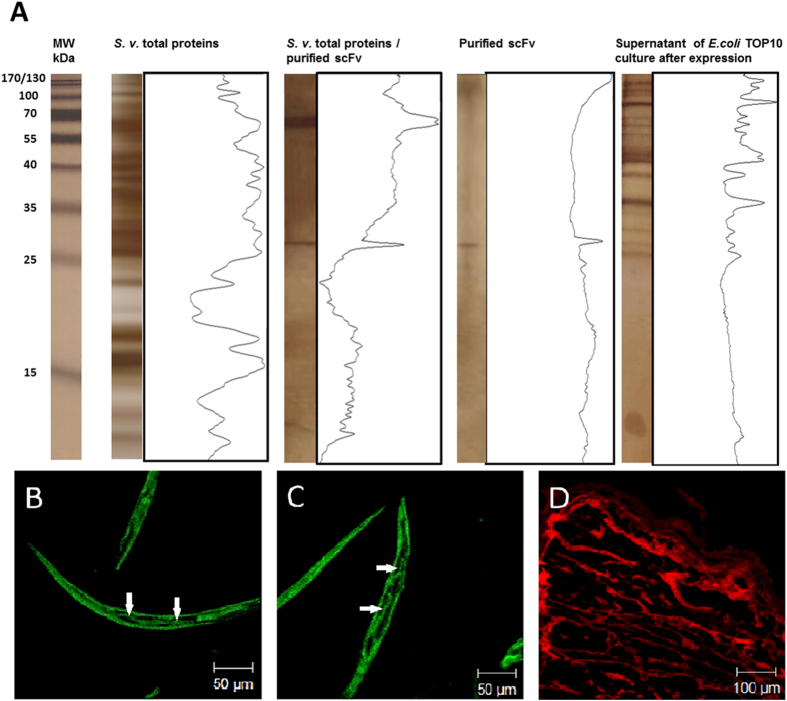
Identification and characterization of the antigenic fraction of *Strongyloides venezuelensis* that bound to the scFv. **** (**A**) Electrophoretic profiles of total proteins detected by silver stained 15% SDS-PAGE after pull-down assay and analyzed by the Image J program. Staining profile of scFv in the body periphery and digestive system (arrow), specifically (**B**) intestine and (**C**) esophagus, from *S. venezuelensis* infective larvae (L3), by immunofluorescence using anti-HA-FITC. (**D**) *Taenia solium* metacestode was used as a negative control of staining. MW: molecular weight standard in kilodaltons (kDa); S. v.: *Strongyloides venezuelensis*; *E. coli*: *Escherichia coli*.

These tests were carried out to identify and characterize the *S. venezuelesis* antigen that was targeted and bound by the scFv. The 15% SDS-PAGE silver stained, after pull-down assay, showed the profiles of the purified scFv (~29 kDa) and the *S. venezuelesis* antigenic fraction of ~65 kDa that bound to the scFv (Fig. 2A). Furthermore, the scFv bound to the body periphery and digestive system (arrow), specifically intestine (Fig. 2B) and esophagus (Fig. 2C), from *S.* *venezuelensis* infective larvae (L3), as evidenced by IFAT. *Taenia solium* metacestodes showed no staining by anti-HA-FITC, but only the red color conferred by counterstaining with 2% Evans blue (Fig. 2D). The antigenic fraction was further stained with Coomassie colloidal blue, trypsinized and characterized by mass spectrometry (CID-MS/MS). A BLAST search showed that this antigenic fraction was a heat shock protein 60 (HSP60) of *Strongyloides* sp. [GenBank:ABY65231.1 and Nematode.net: kq35f01.y1].

### Bioinformatic in silico analysis between scFv and HSP60

**Figure 3:**
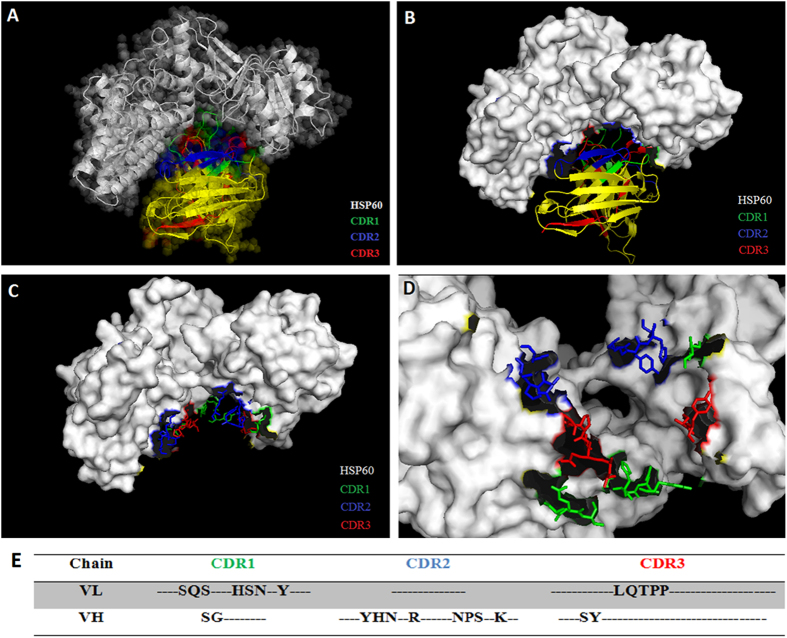
3D structure of scFv binding site on *Strongyloides* sp. HSP60 analyzed by the PyMOL online tool. **** (**A**) Structures demonstrated the CDR regions of the scFv associated to *Strongyloides* sp. HPS60 (GenBank:ABY65231.1). (**B**) Staining of scFv binding site on *Strongyloides* sp. HSP60 and the amino acids of CDR regions that bound to *Strongyloides* sp. HSP60 in both (**C**) frontal and (**D**) apical views. (**E**) Amino acids from CRD regions that bound to the HSP60. CDR: complementarity determining region; HSP: heat shock protein.

Docking between scFv and HSP60 was performed to determine possible binding regions by in silico prediction tools. The most stable 3D structure of the scFv-HSP60 was submitted and analyzed by the PyMOL online tool, and identified the putative CDR regions that are responsible for the antibody binding to the HSP60 epitope with a strong association between them (Fig. 3A, B and C). The binding site analysis also showed that only the CDR2 of the light chain had no binding region to the HSP60 (Fig. 3D,E).

### Sandwich ELISA for immune complexes detection in human sera

**Figure 4:**
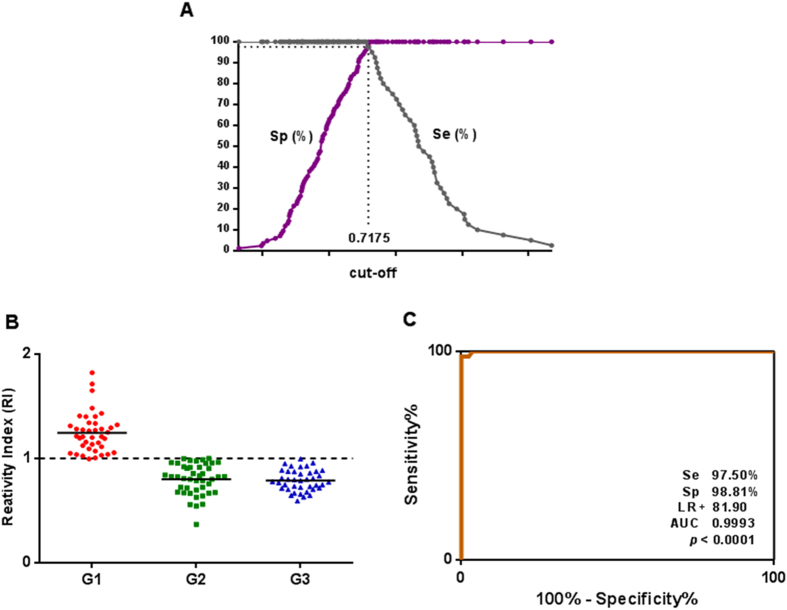
Sandwich ELISA using scFv to detect immune complexes in serum samples. **** (**A**) TG-ROC indicating the optimal cut-off of the method. (**B**) Reactivity indices of sera from individuals with strongyloidiasis (G1, n = 40), other parasitic diseases (G2, n = 44) and healthy individuals (G3, n = 40). (**C**) ROC curve showing sensitivity (Se), specificity (Sp), positive likelihood ratio (LR+) and area under the curve (AUC) (P < 0.001). The horizontal lines separating the data represent the median and dashed line indicates the threshold of the reactivity index.

The scFv was immobilized in high affinity microtiter plates for the serological detection of immune complexes. The TG-ROC curve obtained the best cut-off (0.7175) (Fig. 4A). The data of reactivity index demonstrated a significant discrimination between sera from *S. stercoralis* positive individuals (G1) in relation to serum samples from individuals that were positive for other parasitic diseases (G2) and healthy controls (G3) (P < 0.001) (Fig. 4B). Diagnostic parameters of sensitivity and specificity were 97.5% (86.84 - 99.94) and 98.81% (93.54 - 99.97), respectively. The high efficiency of the test was further demonstrated by the significant positive likelihood ratio (LR+ = 81.90) and area under the curve (AUC = 0.9993) (Fig. 4C).

## Discussion

The engineering of novel functional combinatorial antibodies expressed on the surface of bacteriophages, such as scFvs, with high specificity for antigens coupled with a phage display strategy is the most promising technology for improved diagnostics and therapeutics of many pathologies^10^,^11^. This is due to their small size, great reactivity and specificity, and easiness to maintain and express in bacteria, which surpass the hybridoma technology in all aspects. The scFv selected in this study was structurally characterized and applied in the immune complexes detection using human serum samples positive for strongyloidiasis. This diagnostic strategy is an important tool because it is indicative of active infection^12^,^13^,^14^,^15^.

In this study, we have selected an scFv clone through Phage Display against total proteins of *S. venezuelensis* as a model for *S. stercoralis* infection in humans, with a successful application in the serodiagnosis of human strongyloidiasis. Our novel scFv showed strong reactivity to a parasite protein fraction of ~65 kDa from *S. venezuelensis*, which further identified and characterized to be the heat shock protein 60 (HSP60) from *Strongyloides* sp., a 64 kDa protein shown in experimental models to have relevance to the parasite-host relationship, with potential significance for the host immune response^16^. This newly scFv was localized by immunofluorescence staining in the entire parasite body periphery and to regions of the digestive system from the infective larvae. Moreover, the scFv did not bind to *T. solium* metacestode, indicating specificity.

The scFv functionality was confirmed by a sandwich ELISA using this antibody fragment to detect immune complexes in serum samples from individuals with strongyloidiasis. The TG-ROC curve provided the optimum cut-off value, and ROC curve confirmed its high sensitivity and specificity, both values higher than 97%. The test performance indicated by the high LR+ (81.9) and the AUC value close to the maximum (1.00) conferred a high diagnostic value. The assay was able to discriminate all positive serum samples from patients with strongyloidiasis in relation to sera from healthy individuals and with other parasitic diseases, except one false-positive result for an individual with *Ascaris lumbricoides*, suggesting a possible cross-reaction. However, the very low reactivity indices for the other seven positive sera for *A. lumbricoides* that was below the threshold suggest a possible co-infection of *A. lumbricoides* and *S. stercoralis*, which was not detected by the conventional parasitological method.

To date there have been few studies in the literature employing scFv to diagnose parasitic diseases. The most recent articles have demonstrated its important applicability in the detection of antigenic surface components of *Schistosoma mansoni*^17^ and the selection of antigens for diagnosis of neurocysticercosis^18^. Moreover, there is only one serological assay based on ELISA for the detection of immune complexes in serum samples of individuals with strongyloidiasis^19^, but with a sensitivity of 93.3% and specificity of 86.1%. Unlike this later study, our present investigation used a phage display technology that enabled the *in vitro* production of antibody fragments with high affinity for the target molecule and, consequently, did not require animal models for their synthesis, dramatically reducing the time required to obtain the reagent.

Furthermore, this is the first description of the HSP60 as a diagnostic target for the human strongyloidiasis, with additional data on the epitope and paratope binding sites of the scFv-HSP60 by in silico prediction tools. This result confirms previous studies that described HSPs as major immune dominant antigens in several infections, in addition to their original role as molecular chaperones. It has been demonstrated that HSP60 represents a major component of *Strongyloides ratti* ESPs (excretory/secretory proteins) and presents functions as a target to the humoral and cellular immune response. Interestingly, the vaccination with alum-precipitated HSP60 or a passive immunization with a monoclonal anti-HSP60 IgM antibody, both specifics for *Strongyloides ratti*, conferred protection to mice against a challenge infection^20^,^21^.

This is the first study that has successfully selected and used an scFv biomarker for the diagnosis of human strongyloidiasis, which was applied in the detection of immune complexes in serum samples of patients with great accuracy, aiming the newly found HSP60 as the target antigen. The serological detection of circulating immune complexes of this helminthiasis is indicative of infection, regardless the serum antibody titers and the release of larvae in feces. The development of a better, powerful and innovative diagnostic tool is of public health importance, since strongyloidiasis may be fatal, particularly in immunocompromised individuals.

## Methods

### Ethics statement

All experimental procedures were performed in accordance with the ethical guidelines of the Brazilian Health Ministry, and approved in 2013 (protocol number 307.605) by the Research Ethics Committee from the Federal University of Uberlândia (CEP-UFU), State of Minas Gerais, Brazil. Serum samples from individuals were obtained at the Clinics’ Hospital of the Federal University of Uberlândia (UFU). These samples were based on archived anonymous adult human sera (18–84 years of age) of both genders and were collected in 2009 in the Reference Laboratory for Parasitology Diagnosis at the Federal University of Uberlândia (UFU).

### Serum samples

All serum samples coming from endemic areas for strongyloidiasis obtained from individuals undergoing parasitological stool examinations^22^,^23^ by two independent technical experts in parasitological analysis. These tests were performed with three samples from each individual for parasite identification. Serum samples from 124 individuals were used and divided into 3 groups: Group 1 (G1) – 40 serum samples from individuals infected only with strongyloidiasis; Group 2 (G2) – 44 serum samples from individuals positive by single infection of other intestinal parasitic diseases including *Ascaris lumbricoides* (n = 8), hookworm (n = 7), *Enterobius vermicularis* (n = 5), *Trichuris trichiura* (n = 5), *Schistosoma mansoni* (n = 4), *Hymenolepis nana* (n = 4), *Taenia* sp. (n = 6) and *Giardia lamblia* (n = 5); Group 3 (G3) – 40 serum samples from healthy individuals, based on their clinical observation, without contact or previous infection by *Strongyloides* and three fecal samples tested negative.

### Parasites maintenance and preparation

The *Strongyloides venezuelensis* strain was maintained in Wistar rats (*Rattus norvegiccus*) at the animal research facility (CBEA - Centro de Bioterismo e Experimentação Animal) of UFU. Animals were subcutaneously inoculated with infective larvae (L3). After 7 days of infection, feces were collected for charcoal culture preparation. After 3 days of culture at 27 °C in BOD (Biochemical Oxygen Demand), the L3 were collected^24^, washed 3 times in 0.01 M phosphate-buffered saline (PBS) (pH 7.2), centrifuged at 350 × g for 5 min and stored at -20 °C until use.

### Preparation of total proteins from Strongyloides venezuelensis

*Strongyloides venezuelensis* total proteins were isolated ​​using L3 larvae^25^. Larvae were resuspended in 0.01 M PBS (pH 7.2) containing protease inhibitors and disrupted with five cycles of freezing (1 min, -196 °C) and thawing/sonication (5 min, 40 kHz, 4 °C) (Thornton, Impec Eletrônica, São Paulo, Brazil). After overnight incubation at 4 °C with constant agitation, the suspension was centrifuged at 12,400 × g for 30 min at 4 °C. Protein quantification of the supernatant was performed^26^ and the extract was stored at -20 °C until use.

### Selection of scFvs clones against total proteins of S. venezuelensis

The selection of scFv clones was performed using a human scFv phage library, with approximately 2 × 10^6^ combinatorial sequences with variability for multiple diseases^27^, against total proteins of *S. venezuelensis* as target. Two cycles of selection were performed^18^, being preceded by scFv library reamplification in the competent *Escherichia coli* XL1-Blue strain and infection by VCSM13 helper phage for assembly and replication of viral proteins^28^. A microtiter plate well (Nunc MaxiSorp^TM^) was coated with 50 μL of *S. venezuelensis* total proteins (1 μg/well) diluted in 0.1 M sodium bicarbonate buffer (pH 8.6) and incubated for 18 h at 4 °C. The well was then blocked with 250 μL PBS/0.05% Tween 20 (PBST) (v/v) and 5% BSA (w/v) for 1 h at 37 °C, and washed three times with PBS. Then, 100 μL of the library was added to the well and the plate was incubated for 1 h at 37 °C. Subsequently, the plate was washed 10 times with PBS/0.1% Tween 20, and bound phages to total proteins were eluted with 100 μL of 100 mM glycine-HCl (pH 2.2), followed by neutralization with 16.5 μL of 2 M Tris (pH 9.1). The resulting phages from the first selection were reamplified in *E. coli* XL1-Blue, and a second round of selection was then performed as described above.

### Expression of scFv in E. coli TOP 10

Plasmids were extracted from *Escherichia coli* XL1-Blue bacterial cells infected in the 2nd cycle of selection for further transformation in *E. coli* TOP 10, a non-suppressor strain^28^. Aliquots of the transformed cells were plated on LB (Luria-Bertani) agar containing carbenicillin (100 μg/mL). Plates were incubated at 37 °C for 16 h to allow growth of recombinant colonies. Each colony was transferred to a well in a deep well plate containing 1 mL of Super broth (SB) medium containing carbenicillin (100 μg/mL) and 2% 2 M glucose (v/v). After centrifugation, the expression of soluble scFv was induced by incubation with isopropyl β-D-thiogalactopiranoside (IPTG) (Sigma, USA) to a final concentration of 2.5 mM and carbenicillin (100 μg/mL) overnight at 250 × g and 30 °C. The plates were centrifuged at 4000 × g for 15 min at 4 °C, and the supernatant containing soluble scFv was transferred to another 96-well plate and stored at 4 °C.^28^.

### Analysis of expression and specificity of scFv clones

For analysis of scFv expression, a microtiter plate was coated with suspensions of 96 scFv clones. For analysis of scFv specificity, total proteins of *S. venezuelensis* (10 μg/mL) were immobilized in a micotiter plate and the suspension of 96 scFv clones was added. In both cases anti-HA (tag) peroxidase conjugated (1:1000) (Roche, Switzerland) was added. Reactions were revealed by addition of substrate (30% H_2_O_2_) and the OPD chromogen (orthophenylenodiamine) diluted in 0.1 M citrate-phosphate (pH 5.0). The optical densities were determined at 492 nm in an ELISA reader (Titertek Plus, Flow Laboratories, USA). An irrelevant scFv clone, from the same library but selected against another target, was used as a negative control.

### DNA sequencing and bioinformatics analysis

The sequencing reactions were performed with the DyEnamic ET Dye Terminator Cycle Sequencing Kit (GE Healthcare Life Sciences, USA) and the following primers: MMB4 (5’- GCT TCC GGC TCG TAT GTT GTG T-3’) for the light chain and MMB5 (5’-CGT TTG CCA TCT TTT CAT AAT C-3’) for the heavy chain. The samples were sequenced using an automatic capillary sequencer (MegaBACE 1000 Genetic Analyzer, Amersham Biosciences, USA), and deduced amino acid sequences were then analyzed by IgBlast at NCBI (www.ncbi.nlm.nih.gov/igblast). Furthermore, sequences were submitted to the Raptor-x (http://raptorx.uchicago.edu/) and PyMOL (http://www.pymol.org/) bioinformatics online tools to obtain and analyze the 3D structure of the scFv molecule.

### Medium-scale production of soluble scFv and purification of selected scFv by HPLC

The expression of the selected clone was produced in a 1000 mL Erlenmeyer cell culture flask, as described above (expression of scFv section). For medium-scale purification, His_6_-tagged scFv fragments were purified by immobilized-metal (Nickel) affinity chromatography (HisTrap^TM^ HP, GE Healthcare Life Sciences, USA) according to the manufacturer’s instructions in a HPLC system (AKTA^TM^ purifier). The purified scFv was concentrated using a 10 kDa Amicon apparatus (Millipore, USA), the protein concentration was determined^26^ and a dot-blot assay of the purified scFv was performed^18^ to confirm the efficiency of the purification process.

### Pull-down assay

To characterize the specific protein target that bound to the scFv from the extract of total proteins of *S. venezuelensis*, 100 μL of mouse anti-His mAb Mag Beads (GenScript Transforming Biology Research, USA) were incubated with 100 μL of purified scFv according to the manufacturer´s instructions. After washing, 100 μL of *S. venezuelensis* total proteins (1000 μg/mL) were added and, after incubation and additional washings, 20 μL of the SDS-PAGE (sodium dodecyl sulfate-polyacrylamide gel electrophoresis) sample buffer were added, and the tube was incubated at 100 °C for 5 min. Proteins were eluted from beads using a magnetic apparatus, and the supernatant was submitted to a 15% SDS-PAGE. Proteins were visualized by silver staining^29^, and the electrophoretic profile was determined by Image J 1.44 (National Institute of Health, Bethesda, EUA).

### Immunofluorescence antibody test (IFAT)

*Strongyloides venezuelensis* larvae were embedded in Tissue-tek (Tissue Freezing Medium, Durham, NC, USA) and frozen at -70 °C. Sections of 2 μm in thickness were cut using a cryomicrotome and placed on microscope slides. The sections were left at room temperature for 30 min to dry. Twenty microliters of scFv (750 μg/mL) was added to each section and the slides were incubated in a humidified chamber at 37 °C for 1 h. The sections were incubated with fluorescein isothiocyanate conjugated (FITC) mouse anti-HA (1:1000) and counterstained with 2% Evans blue for 40  min at 37 °C. Between each step three washes were performed in PBS. The slides were mounted using glycerol/PBS (pH 9.0) and coverslips. The staining profile of the scFv on the larvae was verified using a LSM 510 confocal microscope (Meta, Carl Zeiss, Germany). A section of *Taenia solium* metacestode was used as a negative control for staining.

### Mass spectrometry (CID-MS/MS) and bioinformatic analyses

For mass spectrometry, the pull-down assay was repeated; however, the gel was staining with Comassie blue G-250, and the band related to the protein of *S. venezuelensis* that bound to the scFv was trypsinized and submitted to CID-MS/MS^30^ (Quattro II, Micromass, Manchester, UK). Peptide fragments obtained were submitted and analyzed by BLAST (http://www.ncbi.nlm.nih.gov/BLAST/) in relation to their similarity to proteins of *Strongyloides* sp. (taxid: 6247). Furthermore, the highest similar protein sequence was submitted to bioinformatics online tools. The Raptor-x (http://raptorx.uchicago.edu/) was used to obtain the 3D structure of the protein. For identification of potential binding regions of the protein and scFv it was performed a docking (http://bioinfo3d.cs.tau.ac.il/PatchDock/). The regions of scFv-protein structure were selected and analyzed by PyMOL (http://www.pymol.org/).

### Sandwich ELISA for immune complexes detection in human sera

High affinity microtiter plates (Nunc MaxiSorp^TM^) were incubated with 50 μL of scFv (10 μg/mL) in carbonate bicarbonate buffer (0.06 M, pH 9.6), overnight at 4 °C. The plates were blocked with PBS/5% BSA for 45 min at 37 °C. Serum samples were diluted 1:50 in PBST, added to the wells and the plates were incubated for 45 min at 37 °C. Subsequently, mouse anti-human I gG peroxidase conjugated diluted in PBST (1:10,000) was added to the wells and the plates were incubated for 45 min at 37 °C. Between each step three washes were performed in PBST. The reaction was revealed by adding orthophenylenediamine (OPD) diluted in 0.1 M citrate-phosphate (pH 5.0) and 30% H_2_O_2._ Plates were incubated for 15 min at room temperature, and reactions were stopped by adding 2N H_2_SO_4_. The optical densities (OD) were determined at 492 nm in an ELISA reader (Titertek Plus, Flow Laboratories, USA). The cut-off value was determined by the TG-ROC curve^31^, based on the ELISA results. The ELISA reactivity index (RI) was obtained from the ratio: OD of each sample/cut-off value; RI values > 1 were considered positive.

### Statistical analysis

Statistical analysis was performed using GraphPad Prism 5.0 (GraphPad Software Inc., San Diego, USA). The sensitivity (Se), specificity (Sp), and positive likelihood ratio (LR+) were calculated according to the following formulas: Se (%) = a x 100/(a + c); Sp (%) = d x 100/(b + d); LR + = Se/(1 - Sp) where, a: true positive; b: false positive; c: false negative; d: true negative^32^. The area under the curve (AUC) was obtained from the ROC curve in order to determine the diagnostic accuracy of the method^33^. Values were considered statistically significant when *p* < 0.05 and 95% confidence intervals (CI) were provided for Se, Sp and AUC statistical calculations. The Analysis of Variance (ANOVA) followed by the Tukey post hoc was used to determine differences among groups.

## Author Contributions

M.A.L., F.d.A.A.S., P.T.F., J.M.C.C., L.R.G: Conceived and designed the experiments. M.A.L., F.d.A.A.S., P.T.F: Performed the experiments. M.A.L., F.d.A.A.S., P.T.F., J.M.C.C., L.R.G: Analyzed the data. A.P.C., J.M.C.C., L.R.G: Contributed reagents/materials/analyses tools. M.A.L., J.M.C.C., L.R.G: Wrote the manuscript. All authors reviewed the manuscript.

## Additional Information

**How to cite this article**: Levenhagen, M. A. *et al.* Structural and functional characterization of a novel scFv anti-HSP60 of *Strongyloides* sp. *Sci. Rep.* **5**, 10447; doi: 10.1038/srep10447 (2015).
